# Hepatic Transcriptome Analysis Revealing the Molecular Pathogenesis of Type 2 Diabetes Mellitus in Zucker Diabetic Fatty Rats

**DOI:** 10.3389/fendo.2020.565858

**Published:** 2020-11-24

**Authors:** Chengdong Xia, Xiuli Zhang, Tianshu Cao, Jiannong Wang, Cuidan Li, Liya Yue, Kaifeng Niu, Yicheng Shen, Guannan Ma, Fei Chen

**Affiliations:** ^1^ Xiyuan Hospital, China Academy of Chinese Medical Sciences, Beijing, China; ^2^ China National Center for Bioinformation, Beijing, China; ^3^ CAS Key Laboratory of Genome Sciences & Information, Beijing Institute of Genomics, Chinese Academy of Sciences, Beijing, China; ^4^ University of Chinese Academy of Sciences, Beijing, China; ^5^ Department of Virology, State Key Laboratory of Pathogen and Biosecurity, Beijing Institute of Microbiology and Epidemiology, Beijing, China; ^6^ Key Laboratory of Genomic and Precision Medicine, Beijing Institute of Genomics, Chinese Academy of Sciences, Beijing, China

**Keywords:** type 2 diabetes mellitus (T2DM), molecular pathogenesis, substance metabolism, inflammation, liver injury, ER stress, hepatic transcriptome, Zucker diabetic fatty (ZDF) rats

## Abstract

Around 9% of the adult population in the world (463 million) suffer from diabetes mellitus. Most of them (~90%) belong to type 2 diabetes mellitus (T2DM), which is a common chronic metabolic disorder, and the number of cases has been reported to increase each year. Zucker diabetic fatty (ZDF) rat provides a successful animal model to study the pathogenesis of T2DM. Although previous hepatic transcriptome studies revealed some novel genes associated with the occurrence and development of T2DM, there still lacks the comprehensive transcriptomic analysis for the liver tissues of ZDF rats. We performed comparative transcriptome analyses between the liver tissues of ZDF rats and healthy ZCL rats and also evaluated several clinical indices. We could identify 214 and 104 differentially expressed genes (DEGs) and lncRNAs in ZDF rats, respectively. Pathway and biofunction analyses showed a synergistic effect between mRNAs and lncRNAs. By comprehensively analyzing transcriptomic data and clinical indices, we detected some typical features of T2DM in ZDF rats, such as upregulated metabolism (significant increased lipid absorption/transport/utilization, gluconeogenesis, and protein hydrolysis), increased inflammation, liver injury and increased endoplasmic reticulum (ER) stress. In addition, of the 214 DEGs, 114 were known and 100 were putative T2DM-related genes, most of which have been associated with substance metabolism (particularly degradation), inflammation, liver injury and ER stress biofunctions. Our study provides an important reference and improves understanding of molecular pathogenesis of obesity-associated T2DM. Our data can also be used to identify potential diagnostic markers and therapeutic targets, which should strengthen the prevention and treatment of T2DM.

## Introduction

Type 2 diabetes mellitus (T2DM) is a very common chronic metabolic disorder and mainly arises from insulin resistance, which accounts for about 90% of diabetes mellitus (DM) patients (1–4). According to International Diabetes Federation, in 2019, there were around 463 million patients with DM across the globe, corresponding to 9.09% of the adult population in the world (2). China ranks first among all countries, about 116.4 million patients with DM, accounting for approximately one-tenth of the Chinese population. T2DM is often accompanied by various forms of liver injury (5, 6), which facilitates the deterioration of T2DM (7). T2DM and liver injury interact with each other to form a vicious circle.

To study T2DM pathogenesis, many animal models have been successfully established (7). Among them, male Zucker diabetic fatty (ZDF) rats are widely considered to be a successful animal model of diabetic obesity; this model have been extensively applied in the research of T2DM as it aptly simulates the occurrence and development of obesity-associated T2DM (8). Male ZDF rats carry a spontaneous homozygous mutation in the leptin receptor gene; thus, the symptoms of obesity and insulin resistance appear in them at a young age, gradually developing into hyperglycemia and T2DM with age.

Some ZDF rat model-based “omics” studies on T2DM have been reported in recent years (9, 10). Suh YH et al. reported the downregulation of an antioxidant defense gene cluster and cytochrome P450 gene in the adipose and liver tissues of ZDF rats using a cDNA chip (11); Nawano M et al. performed transcriptomic analyses and reported the downregulation of some glucose utilization-related genes and upregulation of some gluconeogenic genes in the skeletal muscle tissue of ZDF rats (12). Slieker LJ et al. also used the ZDF rat model and found that the expression of two important glucose transporter proteins (GLUT2 in islet *beta* cells and GLUT4 in skeletal muscle cells) were downregulated (13).

Although earlier transcriptomic studies have used the ZDF rat model to discover some novel genes associated with T2DM occurrence and development, comprehensive transcriptomic analyses of the liver tissues of ZDF rats have not yet been conducted. Herein we performed comparative transcriptome analyses of the liver tissues of ZDF and Zucker control lean (ZCL) rats, in addition to clinical indices detection. In total, 214 differentially expressed genes (DEGs) and 104 differentially expressed lncRNAs were identified. Pathway and biofunction analyses revealed a synergistic effect between mRNAs and lncRNAs in the liver tissues of ZDF rats. The 214 DEGs included 114 known and 100 putative T2DM-related genes. Our results could provide an important reference and improve the understanding of the molecular pathogenesis of obesity-associated T2DM.

## Materials and Methods

### Animals and Tissues

A group of male ZDF/Gmi-*fa/fa* rats (n = 5) and their gender- and age-matched ZCL (ZCL/Gmi-*+/fa*) rats (n = 5) were purchased at 6 weeks of age from Beijing Vital River Laboratory Animal Technology Co. Ltd. (Beijing, China). The animals were maintained from weeks 6 to 22 in a P3 room with a 12:12-h light/dark cycle at 20°C–25°C and 60% ± 5% atmospheric humidity. The study was approved by the institutional review board of Xiyuan hospital. All animal manipulations and care were performed 1.5–3.5 h after lights were switched on.

The rats were narcotized with pentobarbital sodium (3%, 0.2 ml/100 g) and sacrificed by abdominal aortic exsanguination at week 22. Serum samples were obtained from the blood by centrifugation (2,000 ×g, 4°C, 10 min) and stored at −80°C. Liver tissues were harvested, immediately frozen in liquid nitrogen, and stored at −80°C. Histopathological analyses were performed by routine staining of the frozen sections with hematoxylin and eosin.

### Clinical Indices

FBG levels were measured using Accu-Chek Sensor Comfort test strips (Roche, Germany). Serum levels of triglyceride, cholesterol, alanine aminotransferase, and glutamic oxaloacetic transaminase were measured using enzymatic methods with an automatic biochemistry analyzer (Hitachi 7020, Tokyo, Japan).

### RNA Extraction, Purification, Library Preparation, and Sequencing

Total RNA was extracted and purified from the frozen liver tissues of two ZDF and two ZCL rats using TRIzol (Life Technologies), according to manufacture instructions. The RNA integrity number was calculated to analyze RNA integrity using an Agilent Bioanalyzer 2100 (Agilent Technologies). Subsequently, poly(A)^+^ mRNA was isolated using the Dynabeads^®^ mRNA Purification Kit (Invitrogen), following manufacturer instructions. We then fragmented and purified RNA, synthesized double-stranded cDNA, and prepared indexed Illumina libraries, as described for RNase H libraries. Finally, the library was sequenced on an Illumina HiSeq 4000 system (Illumina, USA), as per the corresponding user guide.

### Sequencing Data Processing

For each sample, the quality of raw sequence data was assessed using FastQC (https://www.bioinformatics.babraham.ac.uk/projects/fastqc/). Reads with mapping quality < 30 were discarded using SAMtools v1.0 (14). Only paired reads were used for subsequent analyses. Clean reads were then aligned to the UCSC rat reference genome (rn6) using TopHat v2.0.9, with default parameters (15, 16). Cufflinks v2.2.1 was used for assembling transcripts (16, 17). DEGs were identified using Cuffdiff v2.2.1 (16). The GTF annotation file for mRNAs was downloaded from the Ensemble website for transcript assembly and gene annotation and that for long noncoding RNAs was downloaded from the NONCODE v5 website.

### Canonical Pathway and Biofunction Analyses

We analyzed differential biofunctions and canonical pathways by IPA (QIAGEN, USA) using differentially expressed mRNAs (FPKM ≥1, *p* < 0.05, |fold-change| ≥ 2), as well as differentially expressed lncRNA (FPKM ≥ 1, *p* < 0.05, |fold-change| ≥ 2) targeted mRNAs. Considering the observed relative abundance of each gene in the cluster and literature-derived regulation direction, the activation states of the biofunctions and canonical pathways were evaluated using the activation Z-score. Z-score > 0 indicated that the cluster was activated and Z-score < 0 indicated that the cluster was inhibited in ZDF rats.

### Statistical Analysis

Statistical analyses were performed using R software v3.4.2. *p* < 0.05 indicated statistical significance.

## Results

### ZDF Rats Displayed Typical Features of T2DM

**Figure 1:**
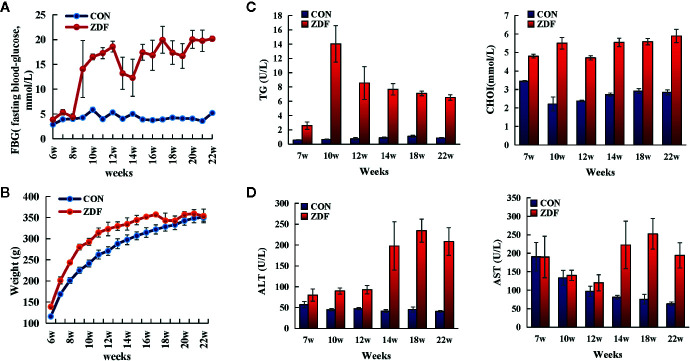
Clinical indices revealing the progression of T2DM in Zucker diabetic fatty (ZDF) rats. Physiological and biochemical indices, including body weight **(A)**, fasting blood glucose **(B)**, serum lipid metabolism indexes **(C)** and hepatic function indexes **(D)**, were constantly monitored in ZDF and healthy ZCL rats from weeks 6 to 22. Five rats were present within each group, and values indicate mean ± SEM. TG, triglycerides; CHOL, total cholesterol; ALT, glutamic pyruvic transaminase; AST, glutamic oxaloacetic transaminase.

To verify the clinical indices of T2DM in male ZDF rats, we measured pertinent physiological and biochemical indices such as body weight, blood glucose levels, blood lipid levels, and serum hepatic marker enzymes (**Figure 1**). Healthy ZCL rats served as controls. Body weight and fasting blood glucose (FBG) levels were constantly monitored from weeks 6 to 22; blood lipid levels and serum hepatic marker enzymes were measured at weeks 7, 10, 12, 14, 18, and 22. In comparison with ZCL rats, ZDF rats showed significantly elevated FBG levels with time, which developed into persistent hyperglycemia from week 9 (**Figure 1A**). Moreover, ZDF rats gained weight from weeks 6 to 17, which reached a plateau at around week 18 (**Figure 1B**). At all measured timepoints, serum triglyceride and serum cholesterol levels in ZDF rats were significantly higher than those in ZCL rats (**Figure 1C**). The aforementioned physiological and biochemical indices demonstrated some typical features of T2DM in ZDF rats (6).

**Figure 2:**
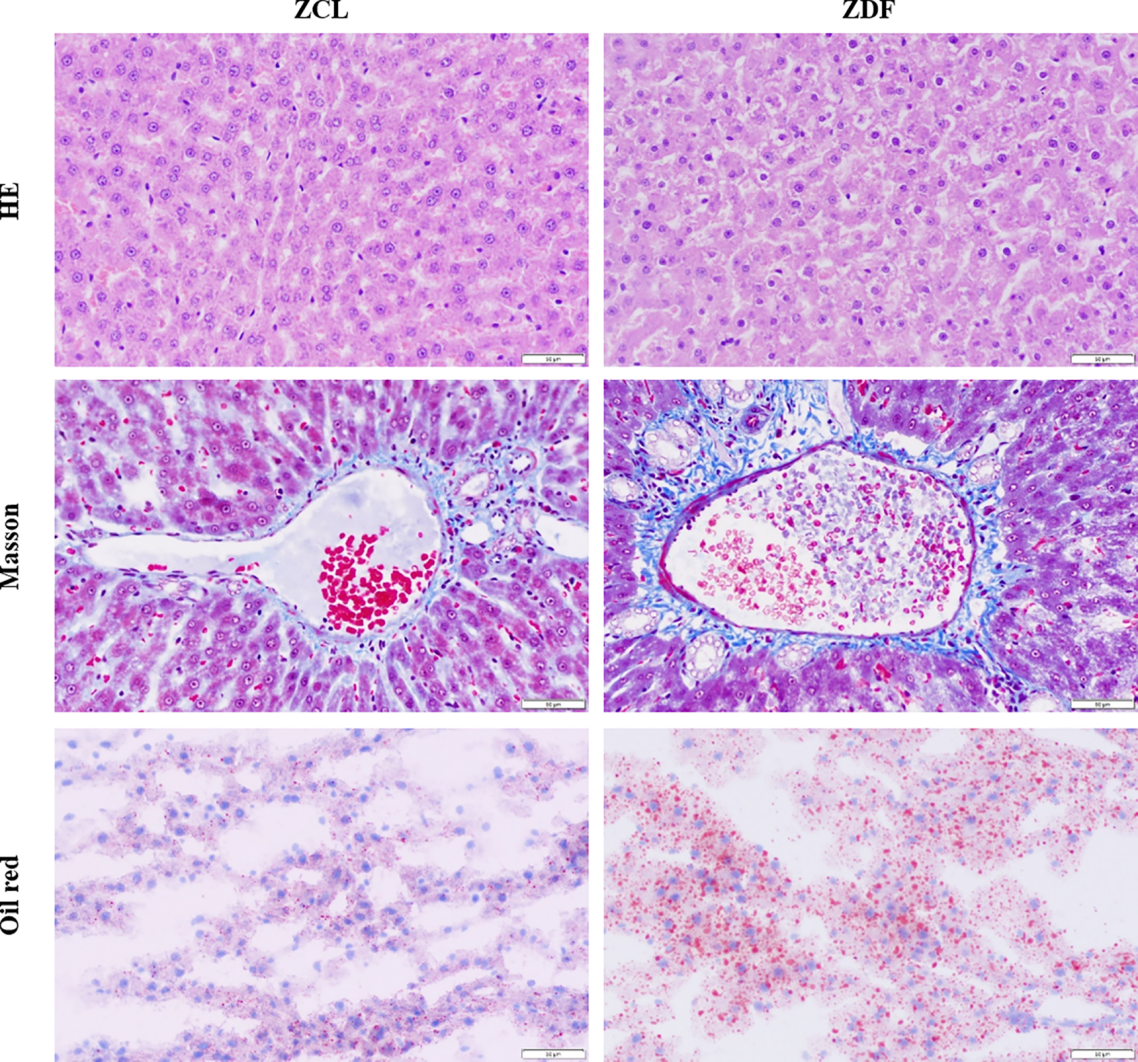
HE, Masson trichrome and Oil red staining of the liver samples from the 22-week-old Zucker diabetic fatty (ZDF) and Zucker control lean (ZCL) rats (Scale bar: 50 µm; 400x).

**Figure 1D** shows that in ZDF rats, the levels of two serum liver enzymes (ALT, glutamic pyruvic transaminase; AST, glutamic oxaloacetic transaminase) were increased, indicating T2DM-induced progressive liver injury. To further verify the presence of liver injury, we performed Hepatic histopathology of 22-week-old ZDF rats and ZCL rats (**Figure 2**). In comparison with ZCL rats, the liver tissue of ZDF rats showed extensive cell turgescence, cytoplasmic degeneration, and narrowed hepatic sinus (**Figure 2**), demonstrating diffuse hepatocyte edema (18). A few hepatic cells with microvesicular steatosis were also observed in the hepatic lobules (**Figure 2**), suggestive of disorders of lipid metabolism (19). These results confirmed the occurrence of liver injury in ZDF rats.

### DEGs and Differentially Expressed lncRNAs in ZDF and ZCL Rats

**Figure 3:**
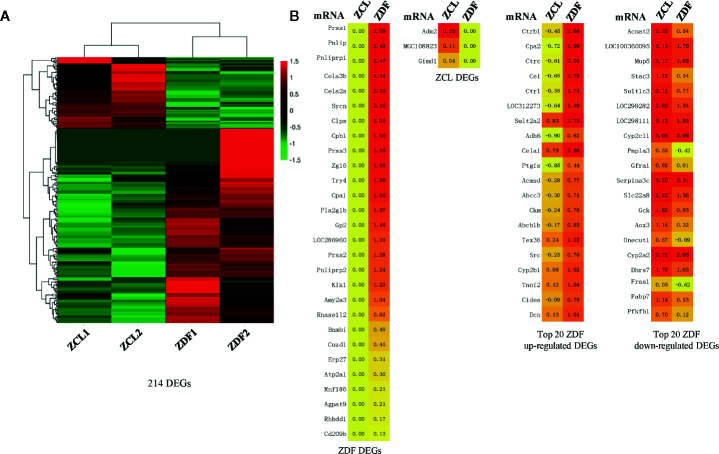
Profiles of differentially expressed mRNAs between Zucker control lean (ZCL) and Zucker diabetic fatty (ZDF) rats. **(A)** Heatmap showing the DEGs between ZCL and ZDF rats (*p* < 0.05, |fold-change| ≥ 2, FPKM ≥ 1). The color key indicates the relative expression level (log10 (FPKM)) between ZCL and ZDF rats, ranging from green (low expression) to red (high expression). **(B)** Heatmaps showing the representative DEGs between ZCL and ZDF rats. Specifically expressed DEGs and top 20 up- and down-regulated DEGs are separately exhibited **(B)**. The colors indicate the expression level (log10 (FPKM)), ranging from yellow (low expression) to red (high expression). The value of are shown in the cells.

Through transcriptomic analyses of liver tissues from 22-week-old ZDF and ZCL rats, we identified 214 DEGs (*p* < 0.05, |fold-change| ≥ 2, FPKM ≥ 1; **Figure 3A**). Among them, 28 DEGs were exclusively expressed in ZDF rats, some of which have been reported to be associated with diabetes, pancreatitis, and pancreatic calcification (**Figure 3B**) (20). On the other hand, three DEGs (Adm2, MGC108823, and Gimd1) were specifically expressed in ZCL rats (**Figure 3B**).

Except the above 31 specifically expressed genes, 183 DEGs (85.5%) were identified to express in both ZDF and ZCL samples. In comparison with ZCL rats, 129 and 54 DEGs were up- and downregulated in ZDF rats, respectively (**Figure 3B**). Among them, 89 upregulated and 24 downregulated DEGs (**Supplementary Table S1**) were found to be associated with diabetes progression (e.g., Ctrb1, Cpa2, and Insig1) and diabetes (e.g., Car3, Dio1, and Mup5), respectively (21, 22).

**Figure 4:**
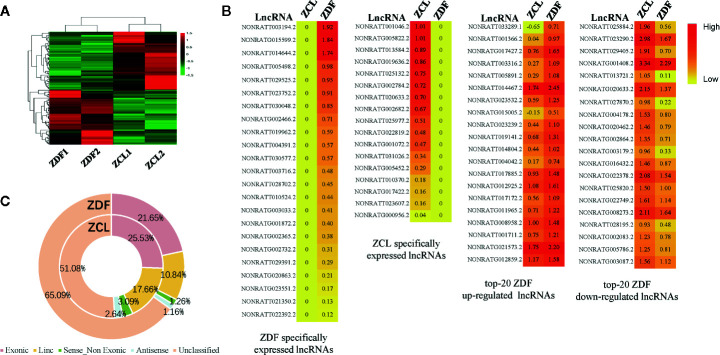
Profiles of differentially expressed lncRNAs between Zucker control lean (ZCL) and Zucker diabetic fatty (ZDF) rats. **(A)** Heatmap showing the differentially expressed lncRNAs between ZCL and ZDF rats (p < 0.05, |fold-change| ≥ 2, FPKM ≥ 1). The color key indicates the relative expression level (log10 (FPKM)) between ZCL and ZDF rats, ranging from green (low expression) to red (high expression). **(B)** Heatmaps showing the representative differentially expressed lncRNAs between ZCL and ZDF rats. Specifically expressed differentially expressed lncRNAs and top 20 up- and down-regulated DEGs are separately exhibited **(B)**. The colors indicate the expression level (log10 (FPKM)), ranging from yellow (low expression) to red (high expression). The value of are shown in the cells. **(C)** The proportions of lncRNA types between ZCL and ZDF rats. The lncRNAs were classified into five types based on the position in the genome, including exonic, lincRNA, sense non-exonic, antisense and unclassified lncRNAs. Each type of circRNA is represented by the indicated color box.

We then analyzed lncRNAs as they have been previously linked to diabetes (23). As shown in **Figure 4**, 104 differentially expressed lncRNAs (*p* < 0.05, |fold-change| ≥ 2, FPKM ≥ 1) were identified; of them, 23 and 17 lncRNAs were specifically expressed in ZDF and ZCL rats, respectively. The remaining 64 lncRNAs were common; 30 and 34 lncRNAs were up- and downregulated in ZDF rats, respectively (**Figures 4A, B**). In addition, according to the genomic position of lncRNAs, we classified them into five types: exonic, lincRNA, sense non-exonic, antisense and unclassified lncRNAs (**Figure 4C**) (24). The results indicated the differential distribution of lncRNA-types between the ZDF and ZCL groups. In comparison with ZCL rats, the proportion of four types of lncRNAs (exonic, linc, sense non-exonic, and antisense) was lower in ZDF rats.

### Pathway and Biofunction Analyses of DEGs and Differentially Expressed lncRNAs

**Figure 5:**
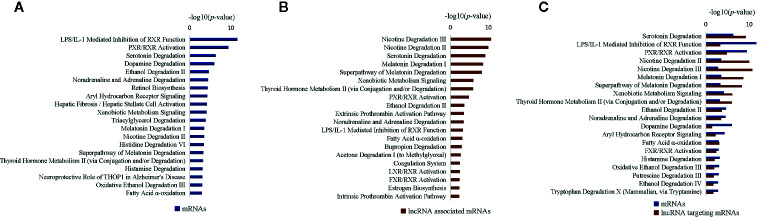
Most significantly affected canonical pathways of differentially expressed genes (DEGs) between Zucker control lean (ZCL) and Zucker diabetic fatty (ZDF) rats. **(A)** Top 20 most significantly affected IPA canonical pathways of differentially expressed mRNAs. **(B)** Top 20 most significantly affected IPA canonical pathways of mRNAs targeted by differentially expressed lncRNAs. **(C)** Top 20 most significantly affected IPA canonical pathways of differentially expressed mRNAs, as well as mRNAs targeted by differentially expressed lncRNAs. The y-axis indicates the significance (-log10(*p*-value)>1.3) of DEG-enriched pathways.

To explore the molecular pathogenesis of T2DM, we used liver samples obtained from ZDF rats and employed the interpretative phenomenological analysis (IPA) approach to subject DEGs and differentially expressed lncRNAs to “canonical pathway” and “biofunction” analyses; all significantly enriched items were then screened (*p* < 0.05). We first conducted “canonical pathway” analyses of the 214 DEGs using the IPA approach, identifying 84 enriched canonical pathways (*p* < 0.05). Further analyses of the top 20 significantly enriched canonical pathways revealed the enrichment of hepatic function-associated substance metabolism (particularly degradation) pathways and inflammatory pathways (**Figure 5A**). Sixteen substance metabolism-associated pathways were identified, including 12 degradation-associated pathways (e.g., “serotonin degradation”, “dopamine degradation” and “ethanol degradation II”); four inflammatory pathways were detected (“LPS/IL-1-mediated inhibition of RXR function,” “PXR/RXR activation”, “aryl hydrocarbon receptor signaling” and “hepatic fibrosis/hepatic stellate cell activation”).

We then conducted “canonical pathway” analyses of the 104 differentially expressed lncRNA-targeted mRNAs using the IPA approach. Seventy-nine canonical pathways (*p* < 0.05) were enriched, including 41 hepatic function-associated substance metabolism (specifically degradation) pathways and 38 inflammatory pathways; these results concurred with the aforementioned findings. The top 20 significantly enriched pathway items (**Figure 5B**) comprised 12 substance metabolism-associated pathways (e.g., “nicotine degradation III”, “nicotine degradation II” and “serotonin degradation”) and eight inflammatory pathways (e.g., “PXR/RXR activation”, “LPS/IL-1-mediated inhibition of RXR function” and “LXR/RXR activation”). Among them, 11 pathways were shared with the top 20 pathway items enriched from DEGs (e.g., “LPS/IL-1-mediated inhibition of RXR function”, “PXR/RXR activation” and “serotonin degradation”; **Figure 5C**), indicating a synergistic effect between mRNAs and lncRNAs.

Further, we used the IPA approach for “biofunction” analyses of DEGs; we identified top 5 significantly enriched items belonging to three categories (“molecular and cellular functions”, “physiological system development and function” and “diseases and disorders”; **Table 1**). Moreover, we observed the enrichment of some hepatic function-associated metabolism and inflammatory biofunction items (*p* < 0.05), such as “carbohydrate metabolism,” “hepatic system development and function,” and “inflammatory response.” These findings were further supported by “biofunction” analyses of differentially expressed lncRNA-targeted mRNAs using the IPA approach (**Supplementary Table S2**). Importantly, seven biofunctions were common between DEGs and differentially expressed lncRNA-targeted mRNAs (e.g., “lipid metabolism”, “digestive system development and function” and “organismal injury and abnormalities”), indicating a synergistic effect between mRNAs and lncRNAs.

**Table 1:** Top-5 IPA Bio Function items of each categories using DEGs.

Category	Bio function	*P-value*	#DEGs
Molecular and Cellular Functions	Lipid Metabolism	2.26E-03 - 8.29E-14	67
	Molecular Transport	2.26E-03 - 8.29E-14	68
	Small Molecule Biochemistry	2.26E-03 - 8.29E-14	80
	Carbohydrate Metabolism	1.84E-03 - 1.92E-10	40
	Vitamin and Mineral Metabolism	1.01E-03 - 1.45E-09	31
Physiological System Development and Function	Digestive System Development and Function	1.65E-03 - 1.72E-09	51
	Organismal Development	2.47E-03 - 2.71E-09	85
	Hepatic System Development and Function	1.52E-03 - 3.85E-09	33
	Organ Morphology	2.47E-03 - 3.85E-09	52
	Tissue Morphology	2.47E-03 - 7.70E-09	66
Diseases and Disorders	Inflammatory Response	2.43E-03 - 5.77E-10	66
	Organismal Injury and Abnormalities	2.58E-03 - 5.77E-10	173
	Gastrointestinal Disease	2.50E-03 - 6.15E-	158
	Hepatic System Disease	2.41E-03 - 6.15E-10	97
	Metabolic Disease	2.02E-03 - 6.15E-10	50

### Molecular Pathogenesis Analysis of T2DM in ZDF Rats

**Figure 6:**
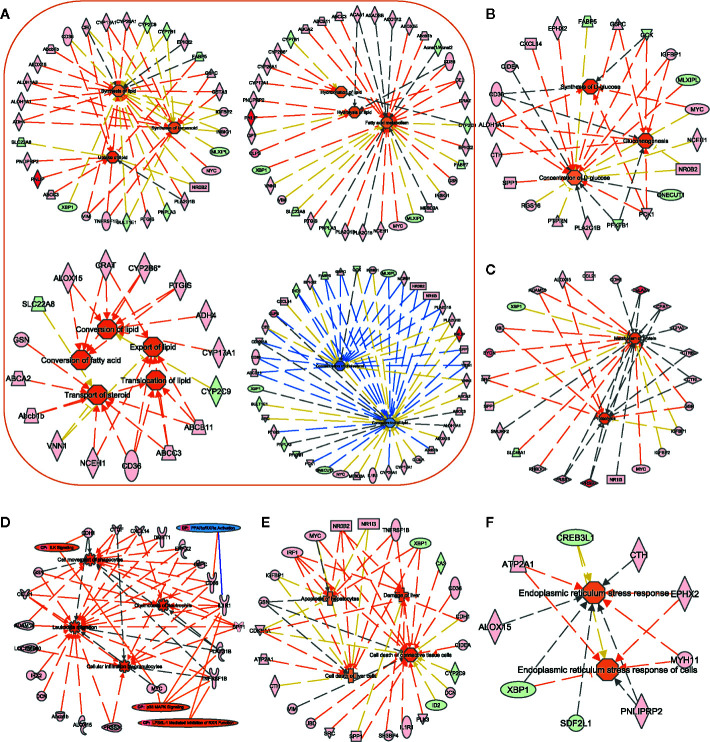
Assessment of the biofunction activity in Zucker diabetic fatty (ZDF) rats. Using the IPA software, we performed functional enrichment analyses of DEGs and evaluated the activation Z-score of the biofunction items, such as “lipid metabolism”- **(A)**, “carbohydrate metabolism”- **(B)**, “protein metabolism”- **(C)**, “immune cell trafficking”- **(D)**, “liver injury”- **(E)**, and “ER stress”- **(F)** related functions. Center icons in networks, significantly activated/inhibited biofunctions; peripheral icons, DEGs between ZCL and ZDF rats and significantly activated/inhibited canonical pathways. Colors of DEGs indicate the estimate of expression levels, (log10 (FPKM)) ranging from green (low expression) to red (high expression). Colors of biofunctions and canonical pathways indicate the activation Z-score, ranging from blue (inhibited) to orange (activated). Orange lines indicate that DEGs support the activation of biofunctions/canonical pathways. Blue lines indicate that DEGs support the inhibition of biofunctions/canonical pathways. Gray lines indicate that DEGs are involved in biofunctions/canonical pathways, but do not affect their activity. DEGs, differentially expressed genes; CP, canonical pathways.

To explore the molecular pathogenesis of T2DM in the liver tissues of ZDF rats, we analyzed all significant differentially expressed canonical pathways and biofunction items using activation Z-score values as well as clinical indices. The comprehensive analysis indicated some typical features of T2DM in ZDF rats, such as abnormal metabolism, increased inflammation, liver injury and increased endoplasmic reticulum (ER) stress (**Figure 6** and **Supplementary Figure S1**).

#### Abnormal Metabolism of Lipid, Glucose, and Protein

In 22-week-old ZDF rats, 94 DEGs were enriched for biofunction items related to lipid, glucose, and protein metabolisms. Most (29 out of 41) lipid metabolism-related biofunctions were activated (**Figure 6A**), such as “uptake of lipid” (Z-score = 1.94), “synthesis of lipid” (Z-score = 0.84), “conversion of fatty acid” (Z-score = 1.95), “hydrolysis of lipid” (Z-score = 1.13), and “export of lipid” (Z-score = 1.56). Correspondingly, “concentration of lipid” (Z-score = −0.82) and “concentration of cholesterol” (Z-score = −1.78) biofunctions were inhibited. These results supported the differences in body weight and hepatic histopathology result between ZDF and ZCL rats at week 22 (**Figures 1B** and **2**).

As for glucose metabolism, “synthesis of D-glucose” (Z-score = 1.72), “gluconeogenesis” (Z-score = 1.62), and “hyperglycemia” (Z-score = 1.67) biofunctions were activated in ZDF rats, and these findings supported the elevation of FBG levels (**Figures 1A** and **6B**).4 protein metabolism-related biofunctions were also activated in ZDF rats, such as “proteolysis” (Z-score = 1.63) and “metabolism of protein” (Z-score = 1.51) (**Figure 6C**). In addition, 78 and 17 lipid/glucose/protein metabolism-related genes were up- and downregulated in ZDF rats, respectively, including glucokinase (GCK), insulin-like growth factor-binding protein-1 (IGFBP1), phosphoenolpyruvate carboxykinase-1 (PCK1), pancreatic lipase (PNLIP), CPA1/2, CTRL, CTRB2, and CELA2A (**Figures 6A–C**, **Supplementary Table S1**).

#### Increased Inflammation

Canonical pathway and biofunction analyses, as well as clinical indices, indicated increased inflammation in ZDF rats. The physiological results revealed the mild infiltration of inflammatory cells in the hepatic lobules and portal area in 22-week-old ZDF rats, indicating increased inflammation (**Figure 2**).

Comparative transcriptome analyses also indicated increased inflammation in ZDF rats upon analyzing related DEGs, canonical pathways, and biofunction items. The expression levels of 92 inflammation response-related DEGs, including EPHX2, G6PC, GSN, SPP1, DMBT1, CDKN1A, ID2, SLC22A8, and SLC7A2, in ZDF rat liver tissues were significantly altered. In addition, 17 inflammation response-related biofunction items were activated (Z-score > 0) in ZDF rats, including the upregulation of some “immune cell trafficking” biofunction items (**Figure 6D**), such as “cell movement of phagocyte” (Z-score = 2.51), “leukocyte migration” (z-score = 2.83), “cellular infiltration by granulocytes” (Z-score = 2.13), and “chemotaxis of neutrophils” (Z-score = 2.41). Moreover, canonical pathway analyses revealed four significantly altered inflammation-related pathways, including “ILK signaling” (Z-score = 2.24), “p38 MAPK signaling” (Z-score = 2.00), “LPS1/IL1-mediated inhibition of RXR function” (Z-score = 0.38), and “PPARα/RXRα activation” (Z-score = −0.45) (**Figure 6D**).

#### Liver Injury

**Figure 6E** shows the upregulation of eight liver injury-related biofunction items, such as “damage of liver” (Z-score = 1.66), “cell death of liver cells” (Z-score = 0.84), and “apoptosis of hepatocytes” (Z-score = 0.82), in liver tissue samples obtained from 22-week-old ZDF rats. Ninety liver injury-related DEGs were also identified, such as ID2, PLK3, IRF1, TNFRSF1B, and XBP1 (**Figure 1D**). This is consistent with the abnormality in serum liver enzymes and pathological observations (**Figures 1D** and **2**).

#### Increased ER Stress

ER stress reportedly can inhibit the insulin signaling pathway, promote insulin resistance, and further accelerate T2DM progression (25). We found that the biofunction “endoplasmic reticulum stress response of cells” (Z-score = 1.08) was upregulated in ZDF rats (**Figure 6F**). Correspondingly, seven (RNF186, MYH11, ALOX15, CTH, PNLIPRP2, ATP2A1, and EPHX2) and three (XBP1, SDF2L1, and CREB3L1) ER stress-related genes were up- and downregulated, respectively, in ZDF rats.

## Discussion

In this study, we performed comparative transcriptome analyses using liver tissue samples, which led to the identification of 214 DEGs and 104 differentially expressed lncRNAs in ZDF and ZCL rats. Pathway and biofunction analyses of DEGs and differentially expressed lncRNAs using the IPA approach indicated a synergistic effect between mRNAs and lncRNAs. Through comprehensive analyses of bioinformatics data and indices, we found some typical features of T2DM in ZDF rats, such as upregulated metabolism (significantly increased lipid absorption/transport/utilization, gluconeogenesis, and protein hydrolysis), increased inflammation, liver injury and increased ER stress.

Among the 214 DEGs, 114 have been reported to be closely related to T2DM occurrence and development (26–30) (**Supplementary Table S3**). Of these 114 DEGs, 83 were found to be involved in T2DM-related substance metabolism; 35 genes were noted to be involved in glycometabolism, 51 in lipid metabolism, and 33 in proteometabolism (**Supplementary Figure S2**). The abnormal expression of GCK, IGFBP1, and PCK1, for example, can cause the occurrence of hyperglycemia (26–28); moreover, the abnormal expression of PNLIP in patients with T2DM has been reported to influence lipid uptake (31) and that of some protein hydrolase/metabolism-related genes (such as CPA1/2, CTRL, CTRB2, and CELA2A) has been reported to be associated with T2DM (21, 29). In addition, in this study, the “ILK signaling” pathway was significantly altered, which is evidently associated with T2DM-related substance metabolism (**Supplementary Figure S1**). As a scaffold, ILK protein can regulate blood glucose levels by recruiting integrin, insulin receptor, and various downstream kinases (32). Of the 114 known T2DM-related DEGs, 65 have been reported to be closely related to some inflammation response-related biofunction items (e.g., “chemotaxis of neutrophils,” “leukocyte migration,” and “cellular infiltration by granulocytes”), such as EPHX2, G6PC, GSN, and SPP1. In addition, four significantly changed pathways were found to be involved in inflammatory responses: upregulation of “ILK signaling” pathway can lead to insulin resistance in T2DM (33) and that of “p38 MAPK signaling” pathway can upregulate immunoinflammatory responses in diabetes by activating cytokines. Moreover, the downregulation of “PPARα/RXRα activation” pathway can enhance inflammatory responses (34–36), and the activation of “LPS1/IL1-mediated inhibition of RXR function” pathway can inhibit the “PPARα/RXRα activation” pathway (**Supplementary Figure S1**), further facilitating the induction of inflammatory responses.

Fifty-eight DEGs of the 114 known T2DM-related DEGs have been reported to be associated with liver injury. IRF1, NR0B2, and TNFRSF1B are related to a significant increase in liver tissue/cell injury (37–39), and the abnormal expression of XBP1 and ATP2A1 can activate some downstream genes (e.g., Bcl2, Bax, and Bim) and cause hepatocyte death (40, 41). Six DEGs of the 114 known T2DM-related DEGs have been reported to be related to ER stress, such as XBP1, MYH11, ATP2A1, EPHX2, and CTH (40–43). We also noted that one ER stress-related pathway was significantly upregulated (“p38 MAPK signaling” pathway): the upregulation of this pathway evidently promotes ER stress and causes insulin resistance (44).

Moving on, we also identified 100 putative T2DM-related DEGs (**Supplementary Figure S2**, **Supplementary Table S4**): 62 were found to be involved in substance metabolism, inflammatory response, liver injury, and ER stress. Moreover, 34 DEGs have been reported to be associated with substance metabolism, such as EEPD1, Abca2, Acnat2, Acot12, Adh4, and Adh6. EEPD1, for instance, could regulate cellular cholesterol levels by upregulating the expression of the cholesterol efflux transporters ABCA1 and ABCG1 (45). **Supplementary Table S1** shows that EEPD1 was specifically expressed in the liver tissue of ZDF rats, suggesting its involvement in T2DM progression.

Of the 100 putative T2DM-associated DEGs, 27 were inflammation response-related DEGs, including DMBT1, CDKN1A (p21), ID2, SLC22A8, and SLC7A2. p21 is an important proinflammatory factor in lymphotoxin-driven pancreatic injury. It could regulate innate immune cell recruitment by influencing the secretion of inflammatory mediators (46).

In addition, we identified 32 liver injury-related genes among the 100 putative T2DM-associated DEGs, including PLK3, CRELD2, DMBT1, FCHSD2, and FNIP2. It is noteworthy that LRH-1 can reportedly induce PLK3 upregulation, leading to the activation of the transcription factor ATF2, consequently causing hepatocyte death (47).

We also realized that four putative T2DM-associated DEGs have been reported to be involved in ER stress (48–51): PNLIPRP2, SDF2L1, ALOX15, and RNF186. The RING finger E3 ligase RNF186 evidently regulates ER stress-mediated apoptosis through interaction with BNip1 in HeLa cells (36, 51–54). As one of the specifically expressed genes in ZDF rats, we deduce that RNF186 plays a key role in T2DM progression.

Interestingly, we found that two significantly enriched disease-associated pathways might be associated with T2DM (“neuroprotective role of THOP1 in Alzheimer’s disease” and “osteoarthritis pathway”) (**Supplementary Figure S1**). A previous proteomic analysis on diabetic retinas (55) revealed that diabetic retinal neurodegeneration shared some common pathogenic pathways with brain neurodegenerative conditions, such as Alzheimer’s and Parkinson’s diseases. Herein we also observed that the “neuroprotective role of THOP1 in Alzheimer’s disease” pathway was upregulated in the liver tissue of ZDF rats [Z-score = 2.45, −log(*p*) = 3.09], suggesting a neuroprotective role in T2DM progression.

Previous research indicated that the increase of inflammatory cytokines could cause insulin resistance by influencing the phosphorylation of IRS in insulin signal transduction (56, 57). This is in agreement with our findings, which showed the increased inflammation in canonical pathways, biofunction items and DEGs for the ZDF samples (**Figure 6D**). On the other hand, abnormal metabolism of glucose is one of the main phenotypes of insulin resistance (56, 57). Our study indicated the higher FBG level in the ZDF samples (**Figure 1A**). Biofunction analysis also showed the activation of “synthesis of D-glucose” (Z-score = 1.72), “gluconeogenesis” (Z-score = 1.62), and “hyperglycemia” (Z-score = 1.67) (**Figure 6B**). Here liver insulin resistance contributes to the excessive HGO, which is highly correlated with hyperglycemia and the increase of FBG level in type 2 diabetes (58).

In addition to insulin resistance, previous studies and our work indicated the phenotypes of obesity and fatty liver in ZDF rats (12) (**Figures 1B** and **2**). Our findings also revealed 15 hepatic steatosis associated DEGs, including CD36, CIDEA, GPT, MFSD2A, MYC, NR0B2, NR1I3, PNLIP, RGS16, XBP1, SULTLE1, FABP5, GCK, PNPLA3 and SPP1 (59–68). Besides, biofunction analysis showed the abnormal metabolism of lipid (**Figure 6A**) and upregulation of liver injury (**Figure 1D**) in ZDF rats. Overall, both phenotype and transcriptome analyses indicated the fatty liver in ZDF rats.

There is a limitation in this study. Our research only study the liver transcriptomes of ZDF rats at one time-point (22-week-old), since they had the stable phenotype of T2DM at that time-point. Certainly, this warrants further study to use more tissues/organs and sampling time-points in the future.

In conclusion, we identified 214 genes that were differentially expressed between ZDF and ZCL rats: 114 known and 100 putative T2DM-related genes. The 114 known T2DM-related genes included 69 substance (glucose/lipid/protein) metabolism-, 65 inflammation response-, 58 liver injury-, 15 hepatic steatosis- and six ER stress-related genes. Further, the 100 putative T2DM-related genes included 25 substance (glucose/lipid/protein) metabolism-, 27 inflammation response-, 32 liver injury-, and four ER stress-related genes. In addition, we observed 84 significantly enriched pathways associated with T2DM progression, including “ILK signaling,” “p38 MAPK signaling,” and “PPARα/RXRα activation” pathways. Our results should serve as an important reference and improve the understanding of obesity-associated T2DM; moreover, the data reported herein should help in the identification of some potential diagnostic markers and therapeutic targets.

## Data Availability Statement

The datasets presented in this study can be found in online repositories. The names of the repository/repositories and accession number(s) can be found below: https://www.ncbi.nlm.nih.gov/geo/, GSE117447.

## Ethics Statement

The animal study was reviewed and approved by Xiyuan hospital, China Academy of Chinese Medical Sciences.

## Author Contributions

FC and GM designed the study. CX, GM, JW, YS, and KN conducted the experiments. XZ, TC, CL, and LY performed the analyses and interpreted the results. FC and XZ wrote the manuscript. All authors contributed to the article and approved the submitted version.

## Funding

This work was supported by the National Natural Science Foundation of China (81373581). The funding body had no role in the design of the study and collection, analysis, and interpretation of data and in writing the manuscript.

## Conflict of Interest

The authors declare that the research was conducted in the absence of any commercial or financial relationships that could be construed as a potential conflict of interest.

## Abbreviations

ALT, alanine aminotransferase; AST, glutamic-oxaloacetic transaminase; CHOL, total cholesterol; DEGs, differentially expressed genes; DM, diabetes mellitus; ER, endoplasmic reticulum; HGO, hepatic glucose output; ILK, integrin linked kinase; IRS, insulin receptor substrate; IPA, Ingenuity Pathway Analysis; lncRNA, long noncoding RNA; LPS, lipopolysaccharide; MAPK, mitogen activated protein kinases; TG, triglycerides; T2DM, type 2 diabetes mellitus; ZDF, Zucker diabetic fatty.
