# Supplementary material for: Hepatic Transcriptome Analysis Revealing the Molecular Pathogenesis of Type 2 Diabetes Mellitus in Zucker Diabetic Fatty Rats
Source: Front Endocrinol (Lausanne). 2020 Nov 24;11:565858. doi: 10.3389/fendo.2020.565858 (PMC7732450; doi:10.3389/fendo.2020.565858)
Supplement: Supplementary file 1 [file DataSheet_1.pdf]

Supplementary Figure S1

Ingenuity Canonical Pathways_mRNAs	z-score	-log(*P*-value)
Neuroprotective Role of THOP1 in Alzheimer's Disease	2.45	3.09
ILK Signaling	2.24	2.01
p38 MAPK Signaling	2.00	1.61
Osteoarthritis Pathway	1.63	2.47
LPS/IL-1 Mediated Inhibition of RXR Function	0.38	11.6
PPARα/RXRα Activation	-0.45	1.59

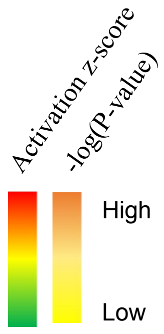

Supplementary Figure S1 Significantly changed canonical pathways of the DEGs between ZDF and ZCL rats using IPA

The left color key represents the activation z-score ranging from low (green) to high (red) (z-score > 0, activation; z-score < 0, inhibition). The right color key indicates the significance ranging from low (yellow) to high (orange) (-log(*p*-value) ＞1.3). The values of z-scores and -log10(*p*-value) are shown in the cells.
