# Supplementary material for: Hepatic Transcriptome Analysis Revealing the Molecular Pathogenesis of Type 2 Diabetes Mellitus in Zucker Diabetic Fatty Rats
Source: Front Endocrinol (Lausanne). 2020 Nov 24;11:565858. doi: 10.3389/fendo.2020.565858 (PMC7732450; doi:10.3389/fendo.2020.565858)
Supplement: Supplementary file 2 [file DataSheet_2.pdf]

Supplementary Figure S2.

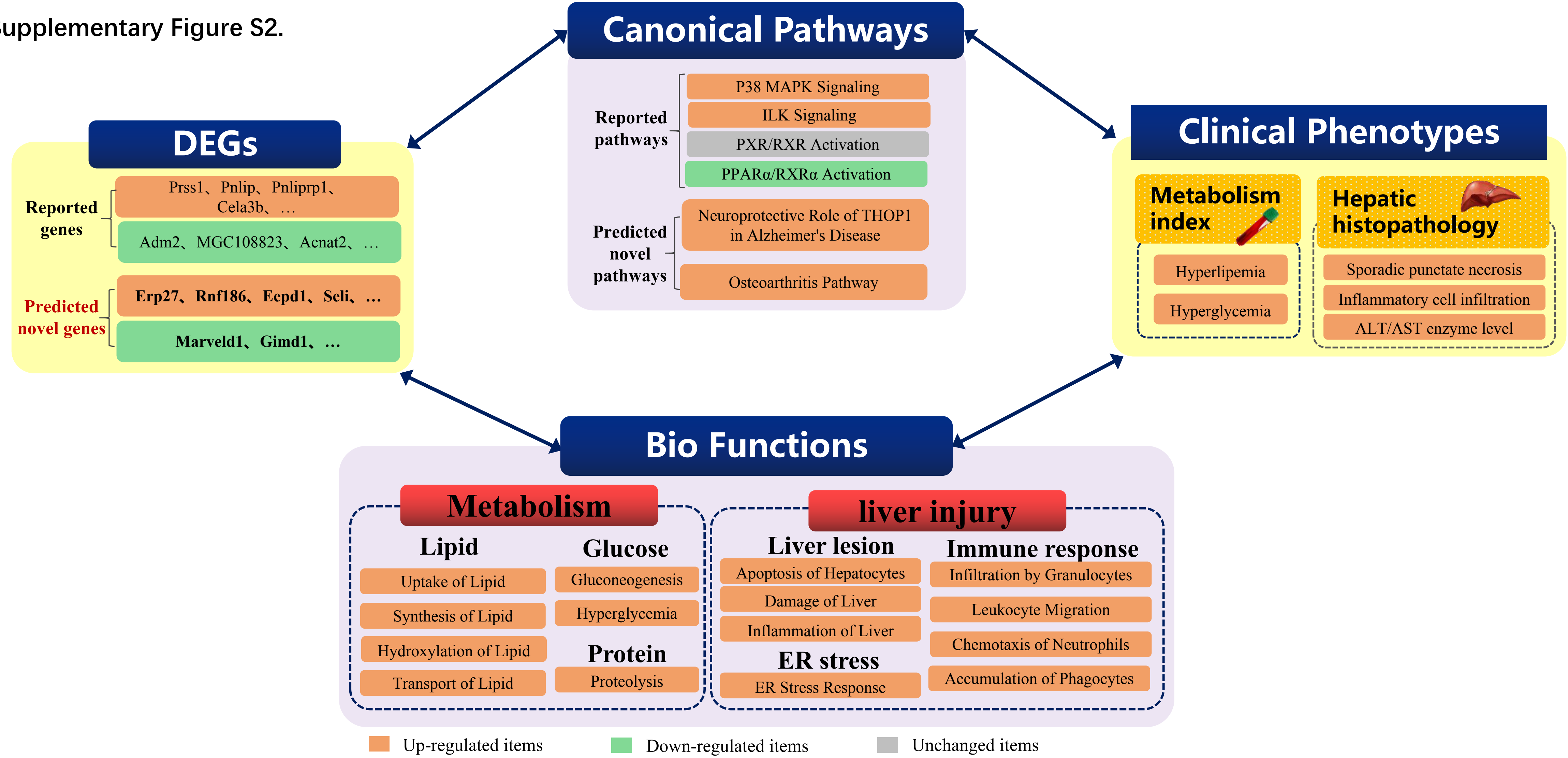

Supplementary Figure S2. Schematic diagram showing the genes/pathways/bioFunctions associated with T2DM

We identified 214 DEGs from the trancriptomic data of liver tissue samples between ZDF and ZCL rats. Among these, 114 DEGs were reported to be associated with T2DM, and 100 DEGs may be possible novel genes. These DEGs were invovled in 84 canonical pathways ($p < 0.05$) and 500 bio function items ($p < 0.05$), among which 6 canonical pathways and 238 bio functions were activated or inhibited ($p < 0.05$, Z-score $\neq 0$). These bio function items and canonical pathways were closely involved in the pathogenesis of typical clinical indices of T2DM (e.g., hyperlipemia, hyperglycemia and liver lesion).
